# Perivascular spaces visible on magnetic resonance imaging predict subsequent delirium in older patients

**DOI:** 10.3389/fnagi.2022.897802

**Published:** 2022-07-18

**Authors:** Quhong Song, Yanli Zhao, Taiping Lin, Jirong Yue

**Affiliations:** Department of Geriatrics and National Clinical Research Center for Geriatrics, West China Hospital, Sichuan University, Chengdu, China

**Keywords:** delirium, perivascular spaces, cerebral small vessel disease, magnetic resonance imaging, older people

## Abstract

**Background:**

It remains unknown whether perivascular spaces (PVS) are associated with delirium in older hospitalized patients. We aimed to determine the association between magnetic resonance imaging (MRI)-visible PVS and the risk of delirium in a cohort of older patients.

**Methods:**

We consecutively recruited older patients (≥70 years) admitted to the Geriatric Department of West China Hospital between March 2016 and July 2017, and their imaging data within one year before admission were reviewed retrospectively. PVS was rated on axial T2-weighted images in the basal ganglia (BG) and centrum semiovale (CS) using the validated semiquantitative 4-point ordinal scale. Delirium was screened within 24 h of admission and three times daily thereafter, using the confusion assessment method. Binary logistic regression analyses were performed to investigate the associations between PVS and delirium.

**Results:**

Among 114 included patients (mean age 84.3 years, 72.8% male), delirium occurred in 20 (17.5%). In patients with MRI examined within 6 months before admission, CS-PVS was found to be associated with delirium (odds ratio [OR] 3.88, 95% confidence interval [CI] 1.07-14.06, unadjusted; and OR 4.24, 95% CI 1.11-16.28, adjusted for age). The associations were enhanced and remained significant even after full adjustment of covariates (OR 7.16, 95% CI 1.16-44.32, adjusted for age, cognitive impairment, smoking, and Charlson Comorbidity Index). Similarly, the relationships between high CS-PVS and delirium were also strengthened after sequentially adjusting all variables of interest, with OR 4.17 (95% CI 1.04-16.73) in unadjusted model and OR 7.95 (95% CI 1.14-55.28) in fully-adjusted model. Adding CS-PVS to the established risk factors improved the risk reclassification for delirium (continuous net reclassification index 62.1%, *P* = 0.04; and integrated discrimination improvement 12.5%, *P* = 0.01).

**Conclusions:**

CS-PVS on MRI acquired 6 months earlier predicts subsequent delirium in older patients and may have clinical utility in delirium risk stratification to enable proactive interventions.

## Introduction

Delirium is a neuropsychiatric syndrome characterized by acute fluctuating disturbances in attention, consciousness, and cognition (Inouye et al., 2014; Marcantonio, 2017). It is common in older hospitalized adults, affecting one-third of those aged 70 years and over (Laurila et al., 2004; Marcantonio, 2017), and is associated with numerous poor outcomes including prolonged hospital stay, cognitive impairment, functional disability, and mortality (Fong et al., 2009; Inouye et al., 2014; Marcantonio, 2017; Goldberg et al., 2020). Though some progress has been made in diagnosis, elucidation of risk factors and prognosis, the neurobiological basis of delirium has not yet been completely clarified. Consequently, few effective and mechanism-based treatments for delirium are available. It is therefore imperative to explore neurobiological biomarkers associated with delirium, which may not only advance our understanding of the pathogenesis of delirium but also aid in delirium risk stratification to implement proactive interventions.

The advancement of neuroimaging particularly magnetic resonance imaging (MRI) provides a unique chance to study the neural substrates of delirium, and certain abnormalities on brain MRI have been reported to be associated with delirium (Nitchingham et al., 2018; Kalvas and Monroe, 2019). Several studies have indicated that white matter hyperintensities, markers of cerebral small vessel disease (CSVD), can predict the occurrence of delirium (Omiya et al., 2015; Nitchingham et al., 2018; Clancy et al., 2021; Pendlebury et al., 2022). Nevertheless, for other CSVD markers, especially MRI-visible perivascular spaces (PVS), this is still unknown. PVS are fluid-filled spaces around the brain perforating small vessels and are hypothesized to act as part of the drainage systems which facilitate fluid exchange and clearance of metabolic wastes from the brain (Wardlaw et al., 2020; Gouveia-Freitas and Bastos-Leite, 2021). Although it is normal to have a few visible PVSs on MRI in healthy young brains (Piantino et al., 2020; Barisano et al., 2021), they may become increasingly common and even enlarge in the context of aging, vascular risk factors, and other features of CSVD (Wardlaw et al., 2020), which may have important clinical implications to brain health (Wardlaw et al., 2020; Gouveia-Freitas and Bastos-Leite, 2021). And growing studies have shown that PVS burden is related to cognitive decline and dementia in older adults (Ding et al., 2017; Debette et al., 2019; Paradise et al., 2021). However, no studies have previously explored PVS in relation to delirium. Whether PVS plays a role in the development of delirium remains unclear.

Therefore, in the present study, we aimed to determine the association between PVS and risk of future delirium and to examine the predictive ability of PVS for delirium in older hospitalized patients. We hypothesized that older patients with a higher PVS burden at baseline would be at greater risk of subsequent delirium.

## Materials and methods

### Study population

Patients in this study were a subsample of our previous cohort study, which prospectively consecutively recruited older internal medical patients admitted to the Department of Geriatric (across four floors), West China Hospital of Sichuan University between March 2016 and July 2017. The details of this cohort have been described previously (Zhao et al., 2021). In brief, eligible patients were aged 70 years and older, with an anticipated stay in the hospital of at least 3 days. Exclusion criteria included delirium on admission, inability to communicate due to severe deafness or severe dementia, terminal conditions with life expectancy <6 months, and incomplete data. A total of 740 patients who met all eligibility criteria were enrolled in this cohort (Supplementary Figure 1), and their imaging data were reviewed retrospectively through our electronic medical systems.

In the present study, patients were further excluded if they did not undergo brain MRI scanning within one year prior to admission or if the MRI image quality was poor, making PVS assessment difficult. This study was approved by the Biomedical Research Ethics Committee of West China Hospital, and conformed to the ethical guidelines of the Declaration of Helsinki. Written informed consent was obtained from all participants.

### Data collection

The following data were collected by trained research nurses within 24 h after admission: age, sex, body mass index (BMI), marital status, education level, smoking, and alcohol drinking. Cognitive function was evaluated using the Short Portable Mental Status Questionnaire (SPMSQ), and errors ≥3 were defined as having cognitive impairment (Pfeiffer, 1975). The severity of comorbidities was rated using the Charlson Comorbidity Index (CCI), a score based on 19 chronic diseases (Charlson et al., 1987). Visual acuity was assessed with the Snellen eye chart, and hearing ability was evaluated with the whispered voice test. Laboratory tests (blood glucose, white blood cell counts, blood urea nitrogen, creatinine, and albumin) were conducted within 24 hours of admission.

### Magnetic resonance imaging acquisition

All included patients underwent brain MRI within the past 1 year prior to this hospital admission using a 3.0 T scanner (Magnetom, Siemens, Erlangen, Germany), with a slice thickness of 5-mm and matrix size of 256 × 256 pixels. MRI sequences included T1-weighted (repetition time [TR] 1600 ms, echo time [TE] 8.6 ms), T2-weighted (TR 4500 ms, TE 105 ms), and fluid-attenuated inversion recovery (FLAIR) images (TR 6000 ms, TE 100 ms).

### Perivascular spaces assessment

The MRI markers were defined and reported following the STandards for ReportIng Vascular changes on nEuroimaging (STRIVE) recommendations (Wardlaw et al., 2013). All MRI images were assessed independently by two trained raters blinded to the patients’ clinical data, and disagreements were resolved by consensus. For patients who underwent multiple MRI imaging during the past 1 year, we used data from the last MRI only.

PVS was defined as fluid-filled compartments surrounding the penetrating vessels, with cerebrospinal fluid (CSF)-like signals on all MRI sequences. They appear linear, round or ovoid, generally smaller than 3 mm in diameter without the surrounding FLAIR hyperintense rim (Wardlaw et al., 2013). PVS was counted on axial T2-weighted images in the basal ganglia (BG) and centrum semiovale (CS), using the validated semiquantitative scale as 0 = none, 1 = 1-10, 2 = 11–20, 3 = 21–40, and 4 ≥40 PVS (Potter et al., 2015a,b). Rating of PVS in the BG was done above the anterior commissure, and included those in the insular cortex (Potter et al., 2015a). For both regions, the slice and side with the most PVS were chosen. High BG-PVS or CS-PVS was defined as a score ≥3, as used previously (Charidimou et al., 2014). The total PVS burden (0-8) was calculated as the sum scores of BG-PVS and CS-PVS. In this study, the interrater weighted kappa for the class of PVS (0-4) was 0.75 for BG-PVS and 0.71 for CS-PVS.

### Other cerebral small vessel disease markers

Lacunes were defined as round or ovoid, subcortical CSF-containing cavities measuring between 3 and 15 mm in diameter with a hyperintense rim on FLAIR (Wardlaw et al., 2013). The presence, number, and location of lacunes were recorded. White matter hyperintensity (WMH) was defined as abnormal hyperintensity of periventricular white matter (PWMH) or deep white matter (DWMH) on FLAIR images. The Fazekas rating scale (a 3-point ordinal scale ranging from 0 to 3) was used to assess the severity of WMH (Fazekas et al., 1987). Extensive WMH was defined as a PWMH score of 3 or DWMH score ≥2. The total WMH burden was calculated by summing the PVWM and DWMH scores. The interrater Cohen-weighted kappa values were 0.65 for lacunes, 0.93 for PWMH, and 0.90 for DWMH.

To assess the cumulative impact of small vessel injury on the brain, we chose lacunes, WMH, and PVS to calculate the “total CSVD burden”. According to the validated ordinal scale (Klarenbeek et al., 2013), one point was allocated if lacune number ≥1, or PVWMH score = 3 and/or DWMH score ≥2, or BG-PVS score ≥2, generating a total score of 0 to 3.

### Prospective assessment of delirium

The main outcome measure was the development of delirium during hospitalization. Delirium was assessed using the confusion assessment method (CAM), a well-validated and standardized assessment tool for delirium that has a high sensitivity (94-100%), specificity (90-95%), and interrater reliability (0.70-1.00) (Wei et al., 2008). The Chinese-language version of the CAM has been used in Mandarin-speaking populations, demonstrating comparable sensitivity and specificity (Gao et al., 2019). The CAM diagnostic algorithm requires the presence of acute onset and fluctuating course, inattention, and either disorganized thinking or an altered level of consciousness to fulfill the criteria for delirium.

Research nurses were trained heavily in screening delirium before the start of the study to ensure high interrater and intrarater reliability (kappa ≥0.9). Then all patients were screened for delirium by well-trained nurses within 24 h after admission and three times daily thereafter until discharge or for a maximum of 13 days, whichever came first. In addition, to minimize misdiagnosis and maximize reliability, experienced clinical researchers further independently assessed patients every 48 h. In case of any doubt, an expert panel was consulted to screen patients according to the Diagnostic and Statistical Manual of Mental Disorders, Fourth Edition criteria (American Psychiatric Association, 2000).

### Statistical analysis

Continuous data were presented as mean with standard deviation (SD) or median with interquartile range (IQR), and categorical data were reported as frequencies and percentages. Intergroup differences in patients with delirium versus those without delirium were detected using the χ^2^ test or Fisher’s exact test for categorical variables and Student’s *t*-test or the Mann-Whitney *U* test for continuous variables.

The binary logistic regression analyses were used to investigate the association between PVS and delirium. Given the limited sample size, only covariates with *P* < 0.05 identified in univariate analysis were included in the multivariate analysis. The following models were generated sequentially: (1) unadjusted; (2) adjusted only for age (model 1); (3) adjusted for age and cognitive impairment (model 2); and (4) adjusted for age, cognitive impairment, smoking, and CCI (model 3). To mitigate the potential bias induced by changes in PVS over time, a similar analysis was performed after excluding patients who underwent MRI more than 6 months before admission.

The receiver operating characteristic (ROC) curve was conducted to evaluate the predictive ability of PVS for delirium. Pairwise comparison of the area under the receiver operating curves (AUCs) was performed using the Delong test (DeLong et al., 1988). In addition, the net reclassification index (NRI) and integrated discrimination improvement (IDI) were further calculated to evaluate the incremental predictive value of PVS beyond conventional risk factors.

All statistical analyses were conducted using Stata 15.0 (StataCorp, College Station, TX, United States) and MedCalc version 20 (MedCalc Software Ltd., Ostend, Belgium). A two-sided *P* < 0.05 was considered to be statistically significant, and multiple comparisons correction was not made due to the exploratory nature of this study.

## Results

### Baseline characteristics

Among 740 patients initially recruited between March 2016 and July 2017, 116 patients underwent brain MRI within the past 1 year, and 2 of those patients with poor MRI quality were further excluded. Therefore, a total of 114 patients were included in the final analysis (Supplementary Figure 1). Compared with the included patients, those excluded patients were younger, had lower education level and lower CCI (all *P* < 0.05, Supplementary Table 1).

Among the 114 included patients, the mean age at admission was 84.3 ± 4.8 years (72.8% male). The median time from MRI scanning to hospital admission was 179 days (IQR, 82-283 days). PVS was detected in all patients, and the median (IQR) PVS scores in the BG and CS were 2 (2-3) and 3 (2-3), respectively. There were twenty (17.5%) patients who experienced delirium during hospitalization. The median time from admission to delirium occurrence was 5 days (IQR, 3-9 days), and the median length of hospital stay was 16 days (IQR 13-25 days). The demographic and clinical characteristics of patients are presented in Table 1. Univariate analysis identified the following characteristics associated with delirium: age, smoking, cognitive impairment, and CCI (all *P* < 0.05); while there was no significant difference in sex, BMI, marital status, education level, alcohol drinking, vision or hearing impairment, or laboratory data.

**TABLE 1:** Demographic and clinical characteristics of study participants.

Characteristics	Total (*n* = 114)	Delirium (*n* = 20)	Non-delirium (*n* = 94)	*P*
Age, mean (SD)	84.3 (4.8)	86.5 (4.4)	83.9 (4.7)	0.03*
Male, n (%)	83 (72.8)	13 (65.0)	70 (74.5)	0.39^†^
BMI, kg/m^2^, mean (SD)	23.5 (3.6)	24.6 (3.8)	23.2 (3.6)	0.13*
Married, n (%)	93 (81.6)	16 (80.0)	77 (81.9)	0.76^‡^
**Education, n (%)**				0.25^‡^
Illiteracy or primary school	7 (6.1)	2 (10.0)	5 (5.3)	
Middle school	20 (17.5)	6 (30.0)	14 (14.9)	
High school and above	87 (76.3)	12 (60.0)	75 (79.8)	
Smoking, n (%)	40 (35.1)	11 (55.0)	29 (30.9)	0.04^†^
Alcohol use, n (%)	18 (15.8)	6 (33.3)	12 (59.3)	0.09^‡^
Blood glucose, mmol/L, mean (SD)	6.60 (2.3)	7.28 (2.8)	6.45 (2.1)	0.14*
WBC, × 10^9^/L, mean (SD)	6.56 (2.6)	7.35 (2.3)	6.39 (2.6)	0.13*
BUN, mmol/l, mean (SD)	7.62 (4.8)	8.10 (3.4)	7.52 (5.0)	0.63*
Creatinine, umol/L, mean (SD)	93.98 (71.5)	75.50 (26.4)	97.91 (77.3)	0.20*
**Primary admission diagnosis, n (%)**				0.56^‡^
AECOPD	19 (16.7)	3 (15.0)	16 (17.0)	
Stroke	15 (13.2)	5 (25.0)	10 (10.6)	
Malignant tumor	12 (10.5)	4 (20.0)	8 (8.5)	
Pneumonia	10 (8.8)	2 (10.0)	8 (8.5)	
Osteoporosis	7 (6.1)	3 (15.0)	4 (4.3)	
Myocardial infarction	6 (5.3)	0 (0.0)	6 (6.4)	
Arrhythmia	6 (5.3)	1 (0.9)	5 (5.3)	
Diabetes mellitus	5 (4.4)	0 (0.0)	5 (5.3)	
Dizziness	5 (4.4)	0 (0.0)	5 (5.3)	
Heart failure	4 (3.5)	0 (0.0)	4 (4.3)	
Hypertension	4 (3.5)	0 (0.0)	4 (4.3)	
Urinary tract infection	3 (2.6)	0 (0.0)	3 (3.2)	
Renal failure	2 (1.8)	0 (0.0)	2 (2.1)	
Others	16 (14.0)	2 (10.0)	14 (14.9)	
Vision impairment, n (%)	37 (32.5)	10 (50.0)	27 (28.7)	0.07^†^
Hearing impairment, n (%)	37 (32.5)	7 (35.0)	30 (31.9)	0.79^†^
Cognitive impairment, n (%)	41 (36.0)	19 (95.0)	22 (23.4)	< 0.001^†^
CCI, median (IQR)	2 (1-3)	2 (2-4)	2 (1-3)	0.01^§^

BMI, body mass index; WBC, white blood cell; BUN, blood urea nitrogen; AECOPD, acute exacerbation of chronic obstructive pulmonary disease; CCI, Charlson comorbidity index; SD, standard deviation; IQR, interquartile range; * t-test; ^†^ χ^2^ test; ^‡^ Fisher’s exact test; ^§^ Mann-Whitney U test.

### Association between perivascular spaces and delirium

**FIGURE 1:**
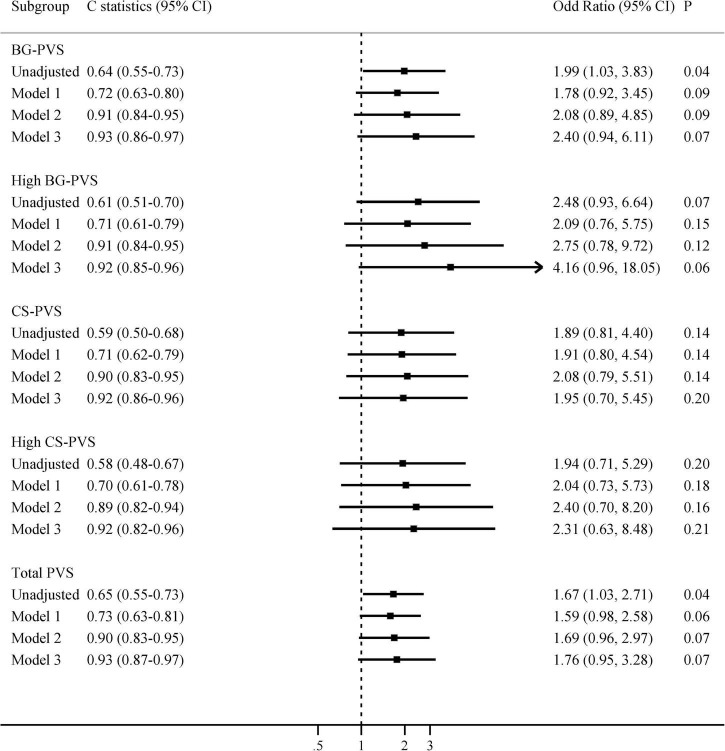
Multivariate associations between PVS and delirium in all patients. Model 1 adjusted for age; Model 2 adjusted for age and cognitive impairment; Model 3 adjusted for age, cognitive impairment, smoking, and Charlson comorbidity index. BG-PVS, basal ganglia perivascular space; CS-PVS, centrum semiovale perivascular space; CI, confidence interval.

The relationships between radiological features and delirium are shown in Table 2 and Supplementary Figure 2. The median BG-PVS score was significantly higher in patients with delirium than in those without delirium (median score 3 vs. 2, *P* = 0.03, Supplementary Figure 2). Delirious patients also had higher PWMH and total WMH scores, while there was no significant difference in CS-PVS, DWMH, or total CSVD burden. In the multivariable analyses, after sequentially adjusting for age, cognitive impairment, smoking and CCI, neither BG-PVS nor CS-PVS was independently associated with delirium (Figure 1).

**TABLE 2:** Imaging characteristics of patients with and without delirium.

Imaging characteristics	Total (*n* = 114)	Delirium (*n* = 20)	Non-delirium (*n* = 94)	*P*
Time from MRI to admission, days, median (IQR)	179 (82-283)	65 (21-202)	188 (107-288)	< 0.01^§^
Presence of lacunes, *n* (%)	24 (21.1)	5 (25.0)	19 (20.2)	0.76^‡^
PWMH, median (IQR)	2 (1-3)	3 (2-3)	2 (1-3)	0.02^§^
Extensive PWMH, *n* (%)	39 (34.2)	10 (50.0)	29 (30.9)	0.10^†^
DWMH, median (IQR)	2 (1-3)	2 (1-3)	1 (1-2)	0.06^§^
Extensive DWMH, *n* (%)	59 (51.8)	13 (65.0)	46 (48.9)	0.19^†^
Total WMH, median (IQR)	4 (2-5)	4 (3-6)	3 (2-5)	0.02^§^
**Severity of total WMH**				0.11^†^
Mild WMH (1-2), *n* (%)	33 (28.9)	2 (10.0)	31 (33.0)	
Moderate WMH (3-4), *n* (%)	43 (37.7)	9 (45.0)	34 (36.2)	
Severe WMH (5-6), *n* (%)	38 (33.3)	9 (45.0)	29 (30.9)	
BG-PVS score, median (IQR)	2 (2-3)	3 (2-3)	2 (2-3)	0.03^§^
**Distribution of BG-PVS burden**				0.14^‡^
Score 1, *n* (%)	21 (18.4)	1 (5.0)	20 (21.3)	
Score 2, *n* (%)	56 (49.1)	9 (45.0)	47 (50.0)	
Score 3, *n* (%)	33 (28.9)	9 (45.0)	24 (25.5)	
Score 4, *n* (%)	4 (3.5)	1 (5.0)	3 (3.2)	
High BG-PVS, *n* (%)	37 (32.5)	10 (50.0)	27 (28.7)	0.07^†^
CS-PVS score, median (IQR)	3 (2-3)	3 (2-3)	2 (2-3)	0.15^§^
**Distribution of CS-PVS burden**				0.46^‡^
Score 1, *n* (%)	8 (7.0)	0 (0.0)	8 (8.5)	
Score 2, *n* (%)	47 (41.2)	7 (35.0)	40 (42.6)	
Score 3, *n* (%)	58 (50.9)	13 (65.0)	45 (47.9)	
Score 4, n (%)	1 (0.9)	0 (0.0)	1 (1.1)	
High CS-EPVS, *n* (%)	59 (51.8)	13 (65.0)	46 (48.9)	0.19^†^
Total PVS score, median (IQR)	5 (4-6)	5 (4-6)	5 (4-5)	0.03^§^
**Distribution of total PVS burden**				0.33^‡^
Score 2, *n* (%)	6 (5.3)	0 (0.0)	6 (6.4)	
Score 3, *n* (%)	17 (14.9)	1 (5.0)	16 (17.0)	
Score 4, *n* (%)	22 (19.3)	4 (20.0)	18 (19.1)	
Score 5, *n* (%)	38 (33.3)	6 (30.0)	32 (43.0)	
Score 6, *n* (%)	30 (26.3)	9 (45.0)	21 (22.3)	
Score 7, *n* (%)	1 (0.9)	0 (0.0)	1 (1.1)	
Total CSVD burden, median (IQR)	2 (1-2)	2 (1-2)	1 (1-2)	0.06^§^
**Distribution of total CSVD burden**				0.26^‡^
Score 0, *n* (%)	11 (9.6)	0 (0.0)	11 (11.7)	
Score 1, *n* (%)	46 (40.4)	7 (35.0)	39 (41.5)	
Score 2, *n* (%)	39 (34.2)	8 (40.0)	31 (33.0)	
Score 3, *n* (%)	18 (15.8)	5 (25.0)	13 (13.8)	

*MRI, magnetic resonance imaging; PWMH, periventricular white matter hyperintensity; DWMH, deep white matter hyperintensity; WMH, white matter hyperintensity; BG-PVS, basal ganglia perivascular space; CS-PVS, centrum semiovale perivascular space; CSVD, cerebral small vessel disease; IQR, interquartile range; ^†^ χ^2^ test; ^‡^ Fisher’s exact test; ^§^ Mann-Whitney U test.*

**FIGURE 2:**
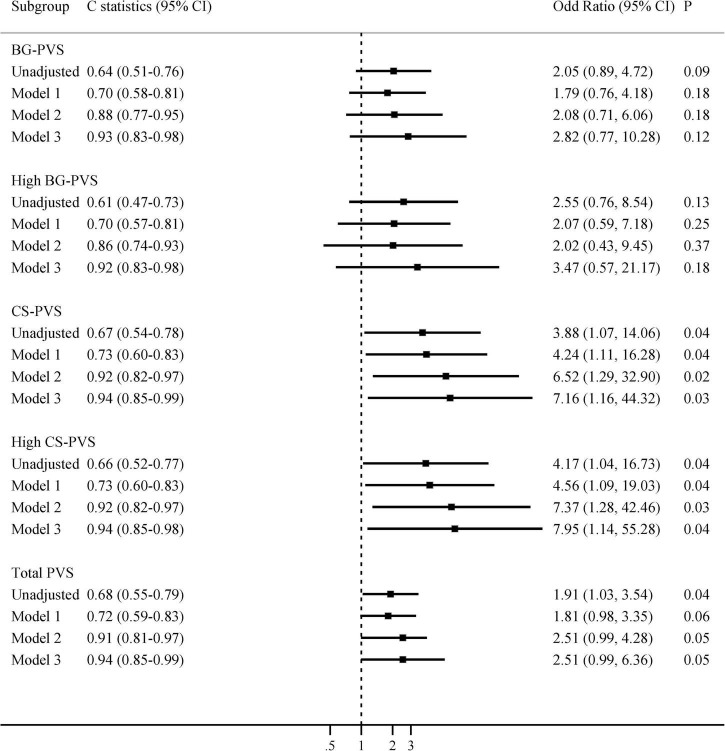
Multivariate associations between PVS and delirium, restricted to patients with MRI examined within the past 6 months. Model 1 adjusted for age; Model 2 adjusted for age and cognitive impairment; Model 3 adjusted for age, cognitive impairment, smoking, and Charlson comorbidity index. BG-PVS, basal ganglia perivascular space; CS-PVS, centrum semiovale perivascular space; CI, confidence interval.

When analyses were restricted to patients with MRI performed ≤6 months prior to hospital admission (*n* = 62), we found that CS-PVS and high CS-PVS were related to delirium in the univariate analysis (median 3 vs. 2, *P* = 0.03; 80.0% vs. 48.9%, *P* = 0.04, Supplementary Table 2). The associations of CS-PVS with delirium were enhanced and remained significant even after full adjustment of covariates: OR 3.88 (95% CI 1.07-14.06) unadjusted, OR 4.24 (95% CI 1.11-16.28) (model 1, adjusted for age), OR 6.52 (95% CI 1.29-32.90) (model 2, adjusted for age and cognitive impairment), and OR 7.16 (95% CI 1.16-44.32) (model 3, adjusted for age, cognitive impairment, smoking and CCI, Figure 2). Similarly, the associations between high CS-PVS and delirium were also strengthened after sequentially adjusting all variables of interest, with an OR of 4.17 (95% CI 1.04-16.73) in the unadjusted model and an OR of 7.95 (95% CI 1.14-55.28) in model 3 **(Figure 2**). In contrast, no associations were seen for PVS in patients with MRI examined >6 months prior to admission (Supplementary Table 3).

Regarding other CSVD markers, additional analyses found that PWMH, DWMH, total WMH, and total CSVD burden were not independently associated with the occurrence of delirium during hospitalization (Supplementary Table 4).

### The predictive ability of perivascular spaces for delirium

**FIGURE 3:**
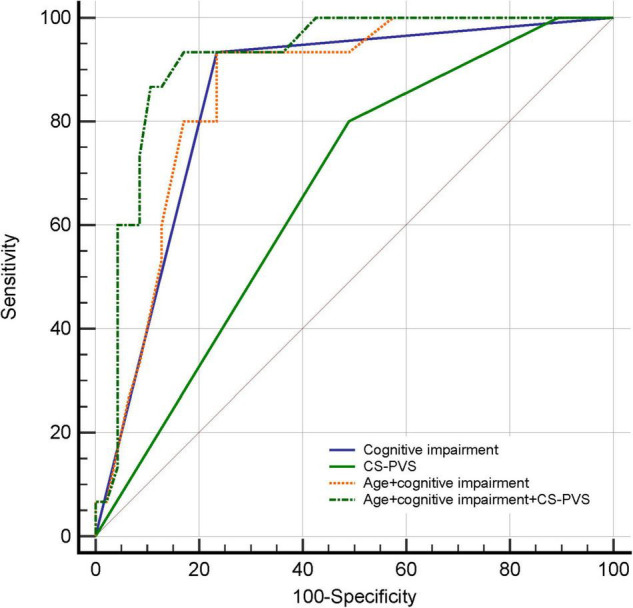
Receiver operating characteristic curves for predicting delirium in patients with MRI examined within the past 6 months. CS-PVS, centrum semiovale perivascular space.

The predictive values of PVS for delirium were summarized in Figures 1, 2 and Supplementary Table 5. The results showed that the predictive ability of BG or CS-PVS for delirium was moderate, but the multivariate models integrating PVS had good discriminatory powers. As CS-PVS burden on MRI acquired within the past 6 months was independently associated with delirium, we then evaluated the incremental predictive value of CS-PVS over the established risk factors for delirium. ROC curve analyses showed an AUC of 0.85 (95% CI 0.74-0.93) for cognitive impairment, 0.67 (95% CI 0.54-0.78) for CS-PVS, and 0.86 (95% CI 0.75-0.94) for the model including age and cognitive impairment (Figure 3). For the model containing age, cognitive impairment and CS-PVS, the AUC was 0.92 (95% CI 0.82-0.97), which was significantly higher than that of cognitive impairment (Delong test, *P* = 0.02) and CS-PVS (Delong test, *P* < 0.001). Although adding CS-PVS to the conventional model containing age and cognitive impairment did not significantly improve discriminatory power (AUC 0.92 vs. 0.86, Delong test *P* = 0.05), the risk reclassification for delirium was significantly improved, with a continuous NRI of 62.1% (*P* = 0.04) and an IDI of 12.5% (*P* = 0.01). Similar results were found for high CS-PVS (Supplementary Table 6).

## Discussion

In the present study, we found that CS-PVS on MRI acquired within 6 months prior to admission was independently associated with subsequent delirium in older hospitalized patients. Our study suggests that existing previously acquired brain MRI may represent an underlying resource, and CS-PVS visible on these MRIs may serve as a risk factor of delirium and may provide important predictive information for delirium occurrence. Incorporating CS-PVS into delirium prediction algorithms might have potential clinical utility in aiding delirium risk stratification, and this needs to be confirmed by future studies.

Although a few studies have investigated brain CSVD markers (e.g., WMH and cerebral microbleeds) in relation to delirium (Omiya et al., 2015; Nitchingham et al., 2018; Lachmann et al., 2019; Clancy et al., 2021; Pendlebury et al., 2022), no previous study has yet explored the link between MRI-visible PVS and delirium. PVS is frequently seen in the aging brain (Bown et al., 2022) and has been proposed as a key contributor to cognitive decline and dementia in older adults (Debette et al., 2019; Paradise et al., 2021). It has been reported that PVS could affect specific cognitive domains, and a negative association of PVS burden with nonverbal reasoning and visuospatial ability has been observed (Maclullich et al., 2004). Recent studies also indicated that PVS was correlated with worse executive function, information processing speed, and mild cognitive impairment (Ding et al., 2017; Passiak et al., 2019; Sepehrband et al., 2021). However, whether PVS plays a role in the development of delirium remains unknown. Our present study adds to evidence on the association of PVS with delirium, suggesting that PVS (particularly that in CS) may be an important risk marker of delirium. The previously reported significant relationship between deep WMH and delirium (Hatano et al., 2013; Omiya et al., 2015) may support our findings regarding PVS in the CS region. However, as the PVS-WMH association has not been fully elucidated (Francis et al., 2019) and the limited studies were available about the correlation of CSVD markers and their distributions with delirium, more investigations are warranted to validate our results and to explore the relationship between PVS locations and delirium. Besides, in this study, we found that the associations between CS-PVS and delirium persisted and remained significant even after adjusting for cognitive impairment, one of the strongest predictors of delirium, which implies that CS-PVS is an independent risk factor for delirium rather than a surrogate measure of cognitive performance.

Several explanations might account for the observed association between CS-PVS and delirium. First, CS-PVS is considered a marker of cerebral amyloid angiopathy (van Veluw et al., 2016; Charidimou et al., 2017). Abnormal protein aggregation (e.g., β-amyloid) can block the upstream within the cortical arteries and impair the external drainage of interstitial fluid in the deep white matter, thus contributing to the retrograde enlargement of PVS in CS (Gouveia-Freitas and Bastos-Leite, 2021), and the relationship between β-amyloid accumulation and CS-PVS has been demonstrated in previous studies (Roher et al., 2003; Charidimou et al., 2015). Furthermore, the impaired perivascular pathway may cause a vicious feed-forward cycle, further prompting PVS dysfunction and β-amyloid deposition in the vascular wall and brain (Gouveia-Freitas and Bastos-Leite, 2021; Bown et al., 2022), which may exacerbate neurovascular dysfunction, neuroinflammation, and neurodegeneration (Carrano et al., 2012; Parodi-Rullan et al., 2021), making the brain vulnerable to delirium (Idland et al., 2017; Maldonado, 2018; Chan et al., 2021).

In addition, PVS contains a range of immune cells contributing to immune surveillance and potentially neuroinflammation (Gouveia-Freitas and Bastos-Leite, 2021). Enlarged PVS is likely to involve inflammation (Wuerfel et al., 2008; Wardlaw et al., 2020) and is proposed as a marker of blood-brain barrier dysfunction (Potter et al., 2015b), which may cause disturbances in the neuronal network and predispose patients to subsequent delirium (Maldonado, 2018). Moreover, it has been reported that metabolite clearance via PVS occurs particularly during sleep (Hablitz et al., 2020; Wardlaw et al., 2020), and sleep deficiency may lead to structural changes in PVS (Del Brutto et al., 2019; Lysen et al., 2021). Growing studies have shown that sleep disruption and circadian dysfunctions, also known contributing factors of delirium (Maldonado, 2018), are involved in the occurrence of enlarged PVS (Berezuk et al., 2015; Del Brutto et al., 2019; Opel et al., 2019; Lysen et al., 2021). These data suggest that the observed relationship between PVS and delirium might be mediated by sleep disturbances. As assessment of sleep-related parameters was not available in the present study, we were unable to explore the potential effect of sleep disturbances. Further studies are warranted to elucidate the complex interrelationships between sleep, PVS, and delirium.

The strengths of our study include that delirium was ascertained at the bedside by trained assessors using the well-validated standardized assessment tool, rather than diagnosed directly from electronic medical records or hospital administrative diagnostic codes (e.g., ICD-10). The rigorous measures of delirium increase the reliability and reduce the misdiagnosis. In addition, this study was the first investigation to explore the association between PVS and delirium. Our study adds new, clinically relevant information to the literature on PVS and delirium, suggesting that CS-PVS on brain MRI could predict delirium occurring up to 6 months later. In other words, our present study suggests that existing previously acquired brain MRIs may represent an underutilized resource, and incorporating neuroimaging markers (e.g., CS-PVS) on these brain MRIs into delirium prediction algorithms may have the potential to identify delirium high-risk patients who may benefit from proactive interventions.

However, some study limitations warrant attention. First, this study was a retrospective analysis based on an internal medicine population in a single geographic area, and our findings may not be generalizable to other populations. Second, the sample size was relatively small and was unequal between delirium and non-delirium groups (the delirium group was 3 times smaller than the non-delirium group), which limited the statistical power and may raise the chance of false positives or false negatives. Besides, given the limited number of outcome events, to avoid overcontrolling, we did not adjust for other potential confounders (such as sensory impairment, treatments after admission, reasons for MRI scans, type and severity of patients’ acute diseases, etc.), and residual confounding cannot be excluded. Our study therefore should be considered preliminary, and future studies with larger sample sizes are needed to validate our findings. Third, correction was not made for multiple comparisons, which may increase the risk of type I error. Our results should be considered as hypothesis generating, and the *P* values should be interpreted with caution. Fourth, potential selection bias may be introduced since many patients without MRI scans were excluded, although there was no significant difference in most baseline characteristics between patients with and those without MRI scans (Supplementary Table 1). Compared to relatively healthier patients who didn’t require MRI, those with MRI might have a lower brain reserve and may have a higher PVS burden, which may potentially affect the relationship between PVS and delirium and limit the generalizability of our results to all older patients. More prospective, multicenter, large-sample studies are warranted to verify our findings. Fifth, as our original cohort was designed to investigate the incidence of delirium, patients with delirium on admission were excluded. Thus, the association between PVS and subsequent delirium observed in this study may be underestimated. In addition, we did not collect data on delirium severity, and whether PVS is related to delirium severity requires further investigation. Finally, PVS was assessed using a semiquantitative visual rating scale. Although practical to use in clinical research with good reliability and repeatability (Potter et al., 2015a), the scale is relatively insensitive and constrained by floor and ceiling effects (Wardlaw et al., 2020; Gouveia-Freitas and Bastos-Leite, 2021). The automated measures of PVS number and volume seem more precise, and further studies are needed to explore their value in predicting delirium.

## Conclusion

CS-PVS on MRI acquired 6 months earlier was independently associated with the occurrence of subsequent delirium in older hospitalized patients. Integration of CS-PVS to the established risk factors enhanced the risk refinement and reclassification for delirium. CS-PVS may serve as a promising marker to identify delirium high-risk patients for proactive implementation of preventive interventions.

## Data Availability Statement

The raw data supporting the conclusions of this article will be made available by the authors, without undue reservation.

## Ethics statement

The studies involving human participants were reviewed and approved by the Biomedical Research Ethics Committee of West China Hospital, and conformed to the ethical guidelines of the Declaration of Helsinki. The patients/participants provided their written informed consent to participate in this study.

## Author contributions

JY and QS conceptualized and designed the study. QS, YZ, and TL collected and interpreted the data. QS analyzed the data, prepared and reviewed the figures, and wrote the original draft. JY provided critical revisions to the manuscript. All authors contributed to the article and approved the submitted version.

## Conflict of Interest

The authors declare that the research was conducted in the absence of any commercial or financial relationships that could be construed as a potential conflict of interest.

## Publisher’s Note

All claims expressed in this article are solely those of the authors and do not necessarily represent those of their affiliated organizations, or those of the publisher, the editors and the reviewers. Any product that may be evaluated in this article, or claim that may be made by its manufacturer, is not guaranteed or endorsed by the publisher.

## References

[B1] American Psychiatric Association (2000). *DSM-IV-TR: Diagnostic and Statistical Manual of Mental Disorders*, 4th Edn. Washington, DC: American Psychiatric Association.

[B2] Barisano G. Sheikh-Bahaei N. Law M. Toga A. W. Sepehrband F. (2021). Body mass index, time of day and genetics affect perivascular spaces in the white matter. *J. Cereb. Blood Flow Metab.* 41 1563–1578. 10.1177/0271678X20972856 33183133 PMC8221772

[B3] Berezuk C. Ramirez J. Gao F. Scott C. J. Huroy M. Swartz R. H. (2015). Virchow-Robin Spaces: Correlations with Polysomnography-Derived Sleep Parameters. *Sleep* 38 853–858. 10.5665/sleep.4726 26163465 PMC4434551

[B4] Bown C. W. Carare R. O. Schrag M. S. Jefferson A. L. (2022). Physiology and Clinical Relevance of Enlarged Perivascular Spaces in the Aging Brain. *Neurology* 98 107–117. 10.1212/WNL.0000000000013077 34810243 PMC8792814

[B5] Carrano A. Hoozemans J. J. van der Vies S. M. van Horssen J. de Vries H. E. Rozemuller A. J. (2012). Neuroinflammation and blood-brain barrier changes in capillary amyloid angiopathy. *Neurodegener Dis.* 10 329–331. 10.1159/000334916 22301467

[B6] Chan C. K. Song Y. Greene R. Lindroth H. Khan S. Rios G. (2021). Meta-analysis of ICU Delirium Biomarkers and Their Alignment With the NIA-AA Research Framework. *Am. J. Crit. Care* 30 312–319. 10.4037/ajcc2021771 34195769 PMC8897570

[B7] Charidimou A. Boulouis G. Pasi M. Auriel E. van Etten E. S. Haley K. (2017). MRI-visible perivascular spaces in cerebral amyloid angiopathy and hypertensive arteriopathy. *Neurology* 88 1157–1164. 10.1212/WNL.0000000000003746 28228568 PMC5373782

[B8] Charidimou A. Hong Y. T. Jager H. R. Fox Z. Aigbirhio F. I. Fryer T. D. (2015). White matter perivascular spaces on magnetic resonance imaging: marker of cerebrovascular amyloid burden? *Stroke* 46 1707–1709. 10.1161/STROKEAHA.115.009090 25908461

[B9] Charidimou A. Jager R. H. Peeters A. Vandermeeren Y. Laloux P. Baron J. C. (2014). White matter perivascular spaces are related to cortical superficial siderosis in cerebral amyloid angiopathy. *Stroke* 45 2930–2935. 10.1161/STROKEAHA.114.005568 25116879

[B10] Charlson M. E. Pompei P. Ales K. L. MacKenzie C. R. (1987). A new method of classifying prognostic comorbidity in longitudinal studies: development and validation. *J. Chronic. Dis.* 40 373–383. 10.1016/0021-9681(87)90171-8 3558716

[B11] Clancy U. Gilmartin D. Jochems A. C. C. Knox L. Doubal F. N. Wardlaw J. M. (2021). Neuropsychiatric symptoms associated with cerebral small vessel disease: a systematic review and meta-analysis. *Lancet Psychiatry* 8 225–236. 10.1016/S2215-0366(20)30431-4 33539776

[B12] Debette S. Schilling S. Duperron M. G. Larsson S. C. Markus H. S. (2019). Clinical Significance of Magnetic Resonance Imaging Markers of Vascular Brain Injury: A Systematic Review and Meta-analysis. *JAMA Neurol.* 76 81–94. 10.1001/jamaneurol.2018.3122 30422209 PMC6439887

[B13] Del Brutto O. H. Mera R. M. Del Brutto V. J. Castillo P. R. (2019). Enlarged basal ganglia perivascular spaces and sleep parameters. A population-based study. *Clin. Neurol. Neurosurg.* 182 53–57. 10.1016/j.clineuro.2019.05.002 31078956

[B14] DeLong E. R. DeLong D. M. Clarke-Pearson D. L. (1988). Comparing the areas under two or more correlated receiver operating characteristic curves: a nonparametric approach. *Biometrics* 44 837–845. 3203132

[B15] Ding J. Sigurethsson S. Jonsson P. V. Eiriksdottir G. Charidimou A. Lopez O. L. (2017). Large Perivascular Spaces Visible on Magnetic Resonance Imaging, Cerebral Small Vessel Disease Progression, and Risk of Dementia: The Age, Gene/Environment Susceptibility-Reykjavik Study. *JAMA Neurol.* 74 1105–1112. 10.1001/jamaneurol.2017.1397 28715552 PMC5695230

[B16] Fazekas F. Chawluk J. B. Alavi A. Hurtig H. I. Zimmerman R. A. (1987). MR signal abnormalities at 1.5 T in Alzheimer’s dementia and normal aging. *Am. J. Roentgenol.* 149 351–356. 10.2214/ajr.149.2.351 3496763

[B17] Fong T. G. Jones R. N. Shi P. Marcantonio E. R. Yap L. Rudolph J. L. (2009). Delirium accelerates cognitive decline in Alzheimer disease. *Neurology* 72 1570–1575. 10.1212/WNL.0b013e3181a4129a 19414723 PMC2677515

[B18] Francis F. Ballerini L. Wardlaw J. M. (2019). Perivascular spaces and their associations with risk factors, clinical disorders and neuroimaging features: A systematic review and meta-analysis. *Int. J. Stroke* 14 359–371. 10.1177/1747493019830321 30762496

[B19] Gao L. L. Feng D. M. Wang R. H. Jiang Y. Y. Zhang M. Yue J. R. (2019). Validity and reliability of the Chinese version of short form of confused assessment method for the detection of delirium in the elderly [in Chinese]. *Shiyong Laonian Yixue.* 33 133–136. 10.3969/j.issn.1003-9198.2019.02.007

[B20] Goldberg T. E. Chen C. Wang Y. Jung E. Swanson A. Ing C. (2020). Association of Delirium With Long-term Cognitive Decline: A Meta-analysis. *JAMA Neurol.* 77 1373–1381. 10.1001/jamaneurol.2020.2273 32658246 PMC7358977

[B21] Gouveia-Freitas K. Bastos-Leite A. J. (2021). Perivascular spaces and brain waste clearance systems: relevance for neurodegenerative and cerebrovascular pathology. *Neuroradiology* 63 1581–1597. 10.1007/s00234-021-02718-7 34019111 PMC8460534

[B22] Hablitz L. M. Pla V. Giannetto M. Vinitsky H. S. Staeger F. F. Metcalfe T. (2020). Circadian control of brain glymphatic and lymphatic fluid flow. *Nat. Commun.* 11:4411. 10.1038/s41467-020-18115-2 32879313 PMC7468152

[B23] Hatano Y. Narumoto J. Shibata K. Matsuoka T. Taniguchi S. Hata Y. (2013). White-matter hyperintensities predict delirium after cardiac surgery. *Am. J. Geriatr. Psychiatry* 21 938–945. 10.1016/j.jagp.2013.01.061 24029014

[B24] Idland A. V. Wyller T. B. Stoen R. Eri L. M. Frihagen F. Raeder J. (2017). Preclinical Amyloid-beta and Axonal Degeneration Pathology in Delirium. *J. Alzheimers Dis.* 55 371–379. 10.3233/JAD-160461 27662296

[B25] Inouye S. K. Westendorp R. G. Saczynski J. S. (2014). Delirium in elderly people. *Lancet* 383 911–922. 10.1016/S0140-6736(13)60688-1 23992774 PMC4120864

[B26] Kalvas L. B. Monroe T. B. (2019). Structural Brain Changes in Delirium: An Integrative Review. *Biol. Res. Nurs.* 21 355–365. 10.1177/1099800419849489 31067980 PMC6794667

[B27] Klarenbeek P. van Oostenbrugge R. J. Rouhl R. P. Knottnerus I. L. Staals J. (2013). Ambulatory blood pressure in patients with lacunar stroke: association with total MRI burden of cerebral small vessel disease. *Stroke* 44 2995–2999. 10.1161/STROKEAHA.113.002545 23982717

[B28] Lachmann G. Kant I. Lammers F. Windmann V. Spies C. Speidel S. (2019). Cerebral microbleeds are not associated with postoperative delirium and postoperative cognitive dysfunction in older individuals. *PLoS One* 14:e0218411. 10.1371/journal.pone.0218411 31199858 PMC6568413

[B29] Laurila J. V. Pitkala K. H. Strandberg T. E. Tilvis R. S. (2004). Detection and documentation of dementia and delirium in acute geriatric wards. *Gen. Hosp. Psychiatry* 26 31–35. 10.1016/j.genhosppsych.2003.08.003 14757300

[B30] Lysen T. S. Yilmaz P. Dubost F. Ikram M. A. de Bruijne M. Vernooij M. W. (2021). Sleep and perivascular spaces in the middle-aged and elderly population. *J. Sleep Res.* 31:e13485. 10.1111/jsr.13485 34549850 PMC9285071

[B31] Maclullich A. M. Wardlaw J. M. Ferguson K. J. Starr J. M. Seckl J. R. Deary I. J. (2004). Enlarged perivascular spaces are associated with cognitive function in healthy elderly men. *J. Neurol. Neurosurg. Psychiatry* 75 1519–1523. 10.1136/jnnp.2003.030858 15489380 PMC1738797

[B32] Maldonado J. R. (2018). Delirium pathophysiology: An updated hypothesis of the etiology of acute brain failure. *Int. J. Geriatr. Psychiatry* 33 1428–1457. 10.1002/gps.4823 29278283

[B33] Marcantonio E. R. (2017). Delirium in Hospitalized Older Adults. *N. Engl. J. Med.* 377 1456–1466. 10.1056/NEJMcp1605501 29020579 PMC5706782

[B34] Nitchingham A. Kumar V. Shenkin S. Ferguson K. J. Caplan G. A. (2018). A systematic review of neuroimaging in delirium: predictors, correlates and consequences. *Int. J. Geriatr. Psychiatry* 33 1458–1478. 10.1002/gps.4724 28574155

[B35] Omiya H. Yoshitani K. Yamada N. Kubota Y. Takahashi K. Kobayashi J. (2015). Preoperative brain magnetic resonance imaging and postoperative delirium after off-pump coronary artery bypass grafting: a prospective cohort study. *Can. J. Anaesth.* 62 595–602. 10.1007/s12630-015-0327-x 25652160

[B36] Opel R. A. Christy A. Boespflug E. L. Weymann K. B. Case B. Pollock J. M. (2019). Effects of traumatic brain injury on sleep and enlarged perivascular spaces. *J. Cereb. Blood Flow Metab.* 39 2258–2267. 10.1177/0271678X18791632 30092696 PMC6827121

[B37] Paradise M. Crawford J. D. Lam B. C. P. Wen W. Kochan N. A. Makkar S. (2021). Association of Dilated Perivascular Spaces With Cognitive Decline and Incident Dementia. *Neurology* 96:e1501–e1511. 10.1212/WNL.0000000000011537 33504642 PMC8032377

[B38] Parodi-Rullan R. M. Javadov S. Fossati S. (2021). Dissecting the Crosstalk between Endothelial Mitochondrial Damage, Vascular Inflammation, and Neurodegeneration in Cerebral Amyloid Angiopathy and Alzheimer’s Disease. *Cells* 10:2903. 10.3390/cells10112903 34831125 PMC8616424

[B39] Passiak B. S. Liu D. Kresge H. A. Cambronero F. E. Pechman K. R. Osborn K. E. (2019). Perivascular spaces contribute to cognition beyond other small vessel disease markers. *Neurology* 92:e1309–e1321. 10.1212/WNL.0000000000007124 30814324 PMC6511092

[B40] Pendlebury S. T. Thomson R. J. Welch S. J. V. Kuker W. Rothwell P. M. (2022). Utility of white matter disease and atrophy on routinely acquired brain imaging for prediction of long-term delirium risk: population-based cohort study. *Age Ageing* 51:afab200. 10.1093/ageing/afab200 34793588 PMC8753040

[B41] Pfeiffer E. (1975). A short portable mental status questionnaire for the assessment of organic brain deficit in elderly patients. *J. Am. Geriatr. Soc.* 23 433–441. 10.1111/j.1532-5415.1975.tb00927.x 1159263

[B42] Piantino J. Boespflug E. L. Schwartz D. L. Luther M. Morales A. M. Lin A. (2020). Characterization of MR Imaging-Visible Perivascular Spaces in the White Matter of Healthy Adolescents at 3T. *AJNR* 41 2139–2145. 10.3174/ajnr.A6789 33033050 PMC7658833

[B43] Potter G. M. Chappell F. M. Morris Z. Wardlaw J. M. (2015a). Cerebral perivascular spaces visible on magnetic resonance imaging: development of a qualitative rating scale and its observer reliability. *Cerebrovasc. Dis.* 39 224–231. 10.1159/000375153 25823458 PMC4386144

[B44] Potter G. M. Doubal F. N. Jackson C. A. Chappell F. M. Sudlow C. L. Dennis M. S. (2015b). Enlarged perivascular spaces and cerebral small vessel disease. *Int. J. Stroke* 10 376–381. 10.1111/ijs.12054 23692610 PMC4463944

[B45] Roher A. E. Kuo Y. M. Esh C. Knebel C. Weiss N. Kalback W. (2003). Cortical and leptomeningeal cerebrovascular amyloid and white matter pathology in Alzheimer’s disease. *Mol. Med.* 9 112–122. 12865947 PMC1430731

[B46] Sepehrband F. Barisano G. Sheikh-Bahaei N. Choupan J. Cabeen R. P. Lynch K. M. (2021). Volumetric distribution of perivascular space in relation to mild cognitive impairment. *Neurobiol. Aging* 99 28–43. 10.1016/j.neurobiolaging.2020.12.010 33422892 PMC7902350

[B47] van Veluw S. J. Biessels G. J. Bouvy W. H. Spliet W. G. Zwanenburg J. J. Luijten P. R. (2016). Cerebral amyloid angiopathy severity is linked to dilation of juxtacortical perivascular spaces. *J. Cereb. Blood Flow Metab.* 36 576–580. 10.1177/0271678X15620434 26661250 PMC4794108

[B48] Wardlaw J. M. Benveniste H. Nedergaard M. Zlokovic B. V. Mestre H. Lee H. (2020). Perivascular spaces in the brain: anatomy, physiology and pathology. *Nat. Rev. Neurol.* 16 137–153. 10.1038/s41582-020-0312-z 32094487

[B49] Wardlaw J. M. Smith E. E. Biessels G. J. Cordonnier C. Fazekas F. Frayne R. (2013). Neuroimaging standards for research into small vessel disease and its contribution to ageing and neurodegeneration. *Lancet Neurol.* 12 822–838. 10.1016/s1474-4422(13)70124-8 23867200 PMC3714437

[B50] Wei L. A. Fearing M. A. Sternberg E. J. Inouye S. K. (2008). The Confusion Assessment Method: a systematic review of current usage. *J. Am. Geriatr. Soc.* 56 823–830. 10.1111/j.1532-5415.2008.01674.x 18384586 PMC2585541

[B51] Wuerfel J. Haertle M. Waiczies H. Tysiak E. Bechmann I. Wernecke K. D. (2008). Perivascular spaces–MRI marker of inflammatory activity in the brain? *Brain* 131 2332–2340. 10.1093/brain/awn171 18676439

[B52] Zhao Y. Yue J. Lei P. Lin T. Peng X. Xie D. (2021). Neutrophil-lymphocyte ratio as a predictor of delirium in older internal medicine patients: a prospective cohort study. *BMC Geriatr.* 21:334. 10.1186/s12877-021-02284-w 34034650 PMC8147036

