# Supplementary material for: Perivascular spaces visible on magnetic resonance imaging predict subsequent delirium in older patients
Source: Front Aging Neurosci. 2022 Jul 18;14:897802. doi: 10.3389/fnagi.2022.897802 (PMC9340666; doi:10.3389/fnagi.2022.897802)

**Supplementary materials**

**Supplementary Table 1** Baseline characteristics between the excluded patients without MRI and the included patients with MRI scanning

| Characteristics | Excluded (n=626) | Included (n=114) | *P* |
| --- | --- | --- | --- |
| Age, mean (SD) | 82.7 (6.2) | 84.3 (4.8) | 0.01^*^ |
| Male, n (%) | 444 (70.9) | 83 (72.8) | 0.66^†^ |
| BMI, kg/m^2^, mean (SD) | 22.8 (3.5) | 23.5 (3.6) | 0.06^*^ |
| Married, n (%) | 517 (82.6) | 93 (81.6) | 0.76^†^ |
| Education, n (%) |  |  | 0.01^†^ |
| Illiteracy or primary school | 104 (16.6) | 7 (6.1) |  |
| Middle school | 113 (18.1) | 20 (17.5) |  |
| High school and above | 409 (65.3) | 87 (76.3) |  |
| Smoking, n (%) | 243 (38.8) | 40 (35.1) | 0.43^†^ |
| Alcohol use, n (%) | 131 (20.9) | 18 (15.8) | 0.21^†^ |
| Blood glucose, mmol/L, mean (SD) | 6.5 (2.9) | 6.6 (2.3) | 0.80^*^ |
| WBC, ×10^9^/L, mean (SD) | 6.9 (3.1) | 6.6 (2.6) | 0.23^*^ |
| BUN, mmol/l, mean (SD) | 7.9 (5.6) | 7.6 (4.8) | 0.64^*^ |
| Creatinine, umol/L, mean (SD) | 100.4 (92.1) | 93.9 (71.5) | 0.48^*^ |
| Vision impairment, n (%) | 206 (32.9) | 37 (32.5) | 0.87^†^ |
| Hearing impairment, n (%) | 192 (30.7) | 37 (32.5) | 0.76^†^ |
| Cognitive impairment, n (%) | 194 (31.0) | 41 (36.0) | 0.29^†^ |
| CCI, median (IQR) | 1 (1-2) | 2 (1-3) | <0.01^§^ |
| Delirium during hospitalization, n (%) | 81 (12.9) | 20 (17.5) | 0.19^†^ |

BMI, body mass index; WBC, white blood cell; BUN, blood urea nitrogen; CCI, Charlson comorbidity index; SD, standard deviation; IQR, interquartile range;

^*^ *t*-test; ^†^ χ^2^ test; ^§^ Mann-Whitney U test.

**Supplementary Table 2** Imaging characteristics of patients with MRI examined within the past 6 months

| Imaging characteristics | Total (n=62) | Delirium (n=15) | Non-delirium (n=47) | *P* |
| --- | --- | --- | --- | --- |
| Presence of lacunes, n (%) | 12 (19.4) | 3 (20.0) | 9 (19.1) | 1.00^‡^ |
| PWMH, median (IQR) | 2 (1-3) | 2 (2-3) | 2 (1-2) | 0.06^§^ |
| Extensive PWMH, n (%) | 17 (27.4) | 6 (40.0) | 11 (23.4) | 0.32^‡^ |
| DWMH, median (IQR) | 1 (1-2) | 2 (1-3) | 1 (1-2) | 0.15^§^ |
| Extensive DWMH, n (%) | 29 (46.8) | 9 (60.0) | 20 (42.6) | 0.24^†^ |
| Total WMH, median (IQR) | 3 (2-5) | 4 (3-6) | 3 (2-4) | 0.06^§^ |
| Severity of total WMH |  |  |  | 0.20^‡^ |
| Mild WMH (1-2), n (%) | 20 (32.3) | 2 (13.3) | 18 (38.3) |  |
| Moderate WMH (3-4), n (%) | 26 (41.9) | 8 (30.8) | 18 (38.3) |  |
| Severe WMH (5-6), n (%) | 16 (25.8) | 5 (33.3) | 11 (23.4) |  |
| BG-PVS score, median (IQR) | 2 (2-3) | 2 (2-3) | 2 (1-3) | 0.07^§^ |
| Distribution of BG-PVS burden |  |  |  | 0.23^‡^ |
| Score 1, n (%) | 13 (21.0) | 1 (6.7) | 12 (25.5) |  |
| Score 2, n (%) | 30 (48.4) | 7 (46.7) | 23 (48.9) |  |
| Score 3, n (%) | 18 (29.0) | 7 (46.7) | 11 (23.4) |  |
| Score 4, n (%) | 1 (1.6) | 0 (0.0) | 1 (2.1) |  |
| High BG-PVS, n (%) | 19 (30.6) | 7 (46.7) | 12 (25.5) | 0.20^‡^ |
| CS-PVS score, median (IQR) | 3 (2-3) | 3 (2-3) | 2 (2-3) | 0.03^§^ |
| Distribution of CS-PVS burden |  |  |  | 0.13^‡^ |
| Score 1, n (%) | 5 (8.1) | 0 (0.0) | 5 (10.6) |  |
| Score 2, n (%) | 22 (35.5) | 3 (20.0) | 19 (40.4) |  |
| Score 3, n (%) | 35 (56.5) | 12 (80.0) | 23 (48.9) |  |
| High CS-PVS, n (%) | 35 (56.5) | 12 (80.0) | 23 (48.9) | 0.04^†^ |
| Total PVS score, median (IQR) | 5 (4-6) | 5 (5-6) | 5 (3-5) | 0.03^§^ |
| Distribution of total PVS burden |  |  |  | 0.37^‡^ |
| Score 2, n (%) | 5 (8.1) | 0 (0.0) | 5 (10.6) |  |
| Score 3, n (%) | 8 (12.9) | 1 (6.7) | 7 (14.9) |  |
| Score 4, n (%) | 11 (17.7) | 2 (13.3) | 9 (19.1) |  |
| Score 5, n (%) | 21 (33.9) | 5 (33.3) | 16 (34.0) |  |
| Score 6, n (%) | 17 (27.4) | 7 (46.7) | 10 (21.3) |  |
| Total CSVD burden, median (IQR) | 1 (1-2) | 2 (1-2) | 1 (1-2) | 0.11^§^ |
| Distribution of CSVD burden |  |  |  | 0.36^‡^ |
| Score 0, n (%) | 6 (9.7) | 0 (0.0) | 6 (12.8) |  |
| Score 1, n (%) | 27 (43.5) | 6 (40.0) | 21 (44.7) |  |
| Score 2, n (%) | 22 (35.5) | 6 (40.0) | 16 (34.0) |  |
| Score 3, n (%) | 7 (11.3) | 3 (20.0) | 4 (8.5) |  |

PWMH, periventricular white matter hyperintensity; DWMH, deep white matter hyperintensity; WMH, white matter hyperintensity; BG-PVS, basal ganglia perivascular space; CS-PVS, centrum semiovale perivascular space; CSVD, cerebral small vessel disease; IQR, interquartile range;

^†^ χ^2^ test; ^‡^ Fisher’s exact test; ^§^ Mann-Whitney U test.

**Supplementary Table 3** Associations between MRI-visible PVS and delirium in patients with brain MRI examined >6 months before hospital admission

|  | Unadjusted |  | Model 1 | | Model 2 |  | Model 3 | |
| --- | --- | --- | --- | --- | --- | --- | --- | --- |
|  | OR (95% CI) | *P* | OR (95% CI) | *P* | OR (95% CI) | *P* | OR (95% CI) | *P* |
| BG-PVS | 2.71 (0.79-9.24) | 0.11 | 2.47 (0.72-8.50) | 0.15 | 2.59 (0.54-12.46) | 0.24 | 2.92 (0.49-17.35) | 0.24 |
| High BG-PVS | 3.20 (0.48-21.21) | 0.23 | 2.74 (0.37-20.07) | 0.32 | 5.18 (0.39-68.53) | 0.23 | 6.44 (0.38-109.37) | 0.20 |
| CS-PVS | 0.54 (0.13-2.34) | 0.41 | 0.52 (0.11-2.41) | 0.41 | 0.45 (0.08-2.42) | 0.35 | 0.38 (0.06-2.41) | 0.31 |
| High CS-PVS | 0.26 (0.03-2.51) | 0.25 | 0.26 (0.03-2.66) | 0.25 | 0.16 (0.01-2.45) | 0.19 | 0.10 (0.004-2.48) | 0.16 |
| Total PVS | 1.34 (0.57-3.18) | 0.50 | 1.30 (0.55-3.07) | 0.56 | 1.12 (0.42-2.96) | 0.83 | 1.10 (0.39-3.06) | 0.86 |

BG-PVS, basal ganglia perivascular space; CS-PVS, centrum semiovale perivascular space; OR, odds ratio; CI, confidence interval;

Model 1: adjusted for age; Model 2: adjusted for model 1 + cognitive impairment; Model 3: adjusted for model 2 + smoking and Charlson comorbidity index.

**Supplementary Table 4** Associations between other CSVD markers and delirium

|  | Unadjusted |  | Model 1 | | Model 2 |  | Model 3 | |
| --- | --- | --- | --- | --- | --- | --- | --- | --- |
|  | OR (95% CI) | *P* | OR (95% CI) | *P* | OR (95% CI) | *P* | OR (95% CI) | *P* |
| **All patients** |  |  |  |  |  |  |  |  |
| PWMH | 2.09 (1.08-4.04) | 0.03 | 1.81 (0.99-3.60) | 0.10 | 1.51 (0.65-3.51) | 0.34 | 1.08 (0.92-1.27) | 0.37 |
| DWMH | 1.77 (1.00-3.12) | 0.05 | 1.53 (0.86-2.75) | 0.15 | 1.27 (0.92-1.24) | 0.41 | 1.38 (0.66-2.98) | 0.39 |
| Total WMH | 1.43 (1.04-1.97) | 0.03 | 1.32 (0.95-1.85) | 0.10 | 1.19 (0.80-1.77) | 0.39 | 1.26 (0.81-1.96) | 0.30 |
| Total CSVD burden | 1.74 (0.98-3.12) | 0.06 | 1.15 (1.00-1.32) | 0.05 | 1.96 (0.88-4.33) | 0.10 | 2.14 (0.92-4.98) | 0.08 |
| **Patients with MRI examined within the past 6 months** | | |  |  |  |  |  |  |
| PWMH | 2.10 (0.95-4.65) | 0.07 | 1.83 (0.80-4.22) | 0.16 | 1.12 (0.38-3.33) | 0.84 | 1.64 (0.47-5.70) | 0.44 |
| DWMH | 1.69 (0.85-3.37) | 0.14 | 1.49 (0.73-3.02) | 0.27 | 1.02 (0.44-2.42) | 0.96 | 1.27 (0.49-3.25) | 0.62 |
| Total WMH | 1.43 (0.96-2.11) | 0.08 | 1.32 (0.88-1.99) | 0.18 | 1.03 (0.62-1.72) | 0.90 | 1.22 (0.68-2.17) | 0.51 |
| Total CSVD burden | 1.90 (0.90-4.01) | 0.09 | 1.75 (0.80-3.82) | 0.16 | 1.97 (0.70-5.52) | 0.20 | 2.49 (0.78-7.88) | 0.12 |
| **Patients with MRI** **examined >6 months before admission** | | | |  |  |  |  |  |
| PWMH | 4.77 (0.70-32.56) | 0.11 | 3.68 (0.52-26.01) | 0.19 | 5.59 (0.60-51.95) | 0.13 | 6.61 (0.69-63.07) | 0.10 |
| DWMH | 3.48 (0.87-13.96) | 0.08 | 2.66 (0.636-11.09) | 0.18 | 3.46 (0.58-20.61) | 0.17 | 3.71 (0.55-25.02) | 0.18 |
| Total WMH | 2.12 (0.91-4.94) | 0.08 | 1.84 (0.78-4.34) | 0.16 | 2.32 (0.77-7.02) | 0.14 | 2.49 (0.80-7.72) | 0.12 |
| Total CSVD burden | 2.17 (0.70-6.70) | 0.18 | 2.39 (0.63-9.02) | 0.20 | 2.81 (0.54-14.56) | 0.22 | 2.81 (0.52-15.21) | 0.23 |

PWMH, periventricular white matter hyperintensity; DWMH, deep white matter hyperintensity; WMH, white matter hyperintensity; CSVD, cerebral small vessel disease; MRI, magnetic resonance imaging; OR, odds ratio; CI, confidence interval;

Model 1: adjusted for age; Model 2: adjusted for model 1 + cognitive impairment; Model 3: adjusted for model 2 + smoking and Charlson comorbidity index.

**Supplementary Table 5** Predictive values of MRI-visible PVS for delirium

|  | AUC (95% CI) | *P* | Cutoff value | Sensitivity (%) | Specificity (%) |
| --- | --- | --- | --- | --- | --- |
| **All patients** |  |  |  |  |  |
| BG-PVS | 0.64 (0.55-0.73) | 0.02 | >2 | 50.00 | 71.28 |
| CS-PVS | 0.59 (0.50-0.68) | 0.11 | >2 | 65.00 | 51.06 |
| Total PVS | 0.65 (0.55-0.73) | 0.04 | >5 | 45.00 | 76.6 |
| High BG-PVS | 0.61 (0.51-0.70) | 0.09 | - | 50.00 | 71.28 |
| High CS-PVS | 0.58 (0.48-0.67) | 0.18 | - | 65.00 | 51.06 |
| **Patients with MRI examined within the past 6 months** | | | |  |  |
| BG-PVS | 0.64 (0.51-0.76) | 0.05 | >2 | 46.67 | 74.47 |
| CS-PVS | 0.67 (0.54-0.78) | 0.007 | >2 | 80.00 | 51.06 |
| Total PVS | 0.68 (0.55-0.79) | 0.01 | >5 | 46.67 | 78.72 |
| High BG-PVS | 0.61 (0.47-0.73) | 0.15 | - | 46.67 | 74.47 |
| High CS-PVS | 0.66 (0.52-0.77) | 0.02 | - | 80.00 | 51.06 |

BG-PVS, basal ganglia perivascular space; CS-PVS, centrum semiovale perivascular space; MRI, magnetic resonance imaging; AUC, area under the receiver operating characteristics curve; CI, confidence interval; The optimal cutoff value was determined by the Youden index.

**Supplementary Table 6** The incremental predictive value of CS-PVS over the established risk factors for delirium among patients with brain MRI examined within the past 6 months

|  | AUC (95% CI) | *P* of Delong test | Continuous NRI, *P* | IDI, *P* |
| --- | --- | --- | --- | --- |
| **CS-PVS** |  |  |  |  |
| CS-PVS | 0.67 (0.54-0.78) | <0.001^a^ | - | - |
| Cognitive impairment | 0.85 (0.74-0.93) | 0.02 ^a^ | - | - |
| Age + cognitive impairment | 0.86 (0.75-0.94) | 0.05 ^a^ | Reference | Reference |
| Age + cognitive impairment + CS-PVS | 0.92 (0.82-0.97) | - | 62.1%, 0.04 | 12.5%, 0.01 |
| **High CS-PVS** |  |  |  |  |
| High CS-PVS | 0.66 (0.52-0.77) | <0.001^a^ | - | - |
| Cognitive impairment | 0.85 (0.74-0.93) | 0.03 ^a^ | - | - |
| Age + cognitive impairment | 0.86 (0.75-0.94) | 0.06 ^a^ | Reference | Reference |
| Age + cognitive impairment + high CS-PVS | 0.92 (0.82-0.97) | - | 62.1%, 0.04 | 10.6%, 0.04 |

CS-EPVS, centrum semiovale perivascular space; AUC, area under the receiver operating curve; NRI, net reclassification index; IDI, integrated discrimination improvement; CI, confidence interval;

^a^ Compared to the three-item model containing age, cognitive impairment and CS-PVS/ high CS-PVS.

**Supplementary Figure 1** Flow chart of participants’ selection

1202 patients admitted to our hospital between March 2016 and July 2017

1. patients excluded:

- Delirium on admission (n=33)
- Unable to communicate due to severe deafness (n=119)
- Unable to communicate due to severe dementia (n=113)
- Terminal condition (n=107)
- Incomplete data (n=90)

740 patients included in the original cohort

626 patients further excluded:

- No brain MRI within the past 1 year (n=624)
- Poor MRI quality (n=2)

114 patients included in the final analysis

50 patients experienced delirium during hospitalization

437 patients did not experience delirium during hospitalization

50 patients experienced delirium during hospitalization

437 patients did not experience delirium during hospitalization

50 patients experienced delirium during hospitalization

437 patients did not experience delirium during hospitalization

62 patients with MRI examined within the past 6 months

52 patients with MRI examined >6 months before admission

**Supplementary Figure 2** Distribution of PVS burden between delirium and non-delirium groups in all patients (BG-PVS: **A, D**; CS-PVS: **B, E**; total PVS: **C, F**)


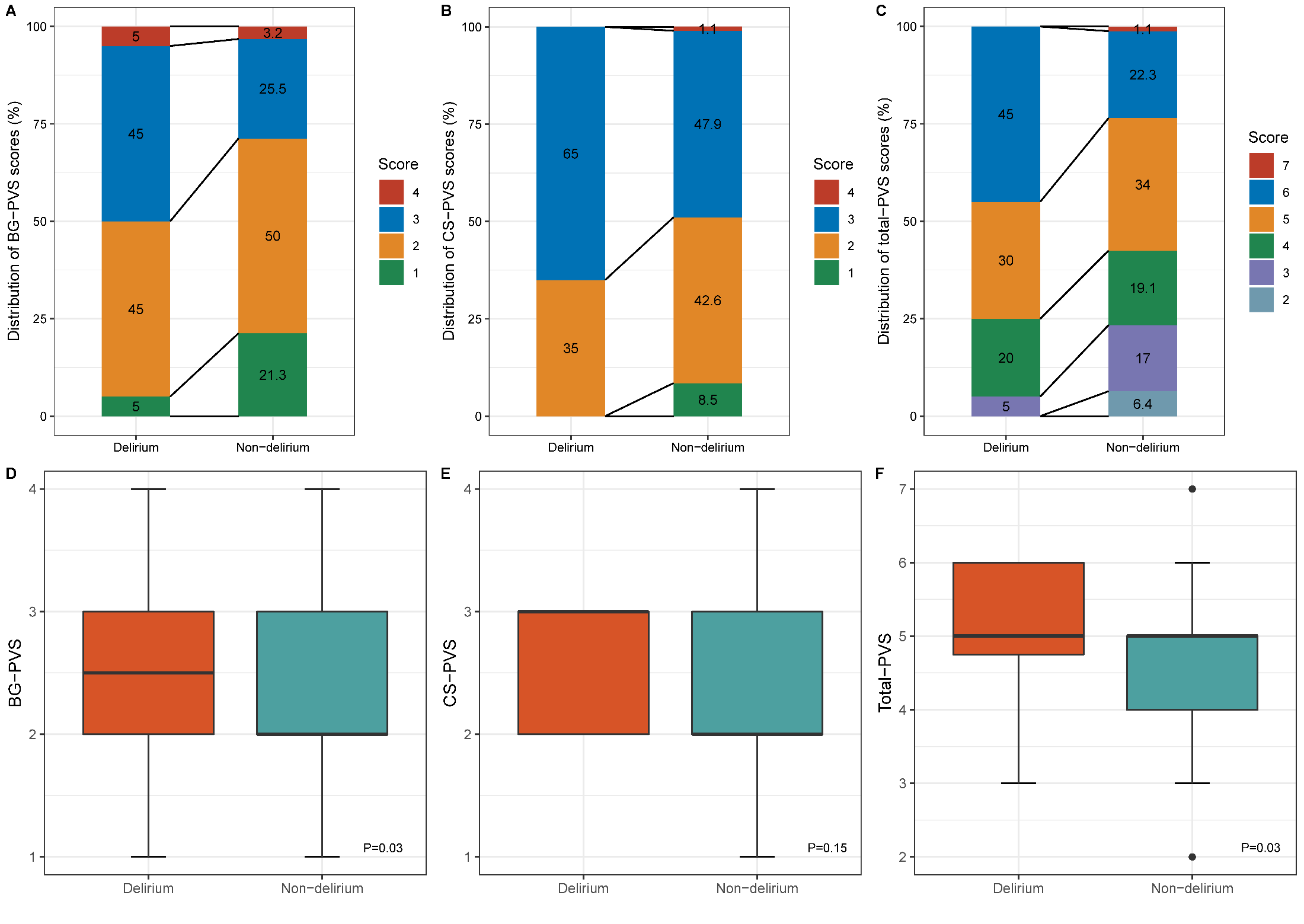


**Supplementary Figure 3** Distribution of PVS burden between delirium and non-delirium groups in patients with MRI examined within the past 6 months (BG-PVS: **A, D**; CS-PVS: **B, E**; total PVS: **C, F**)


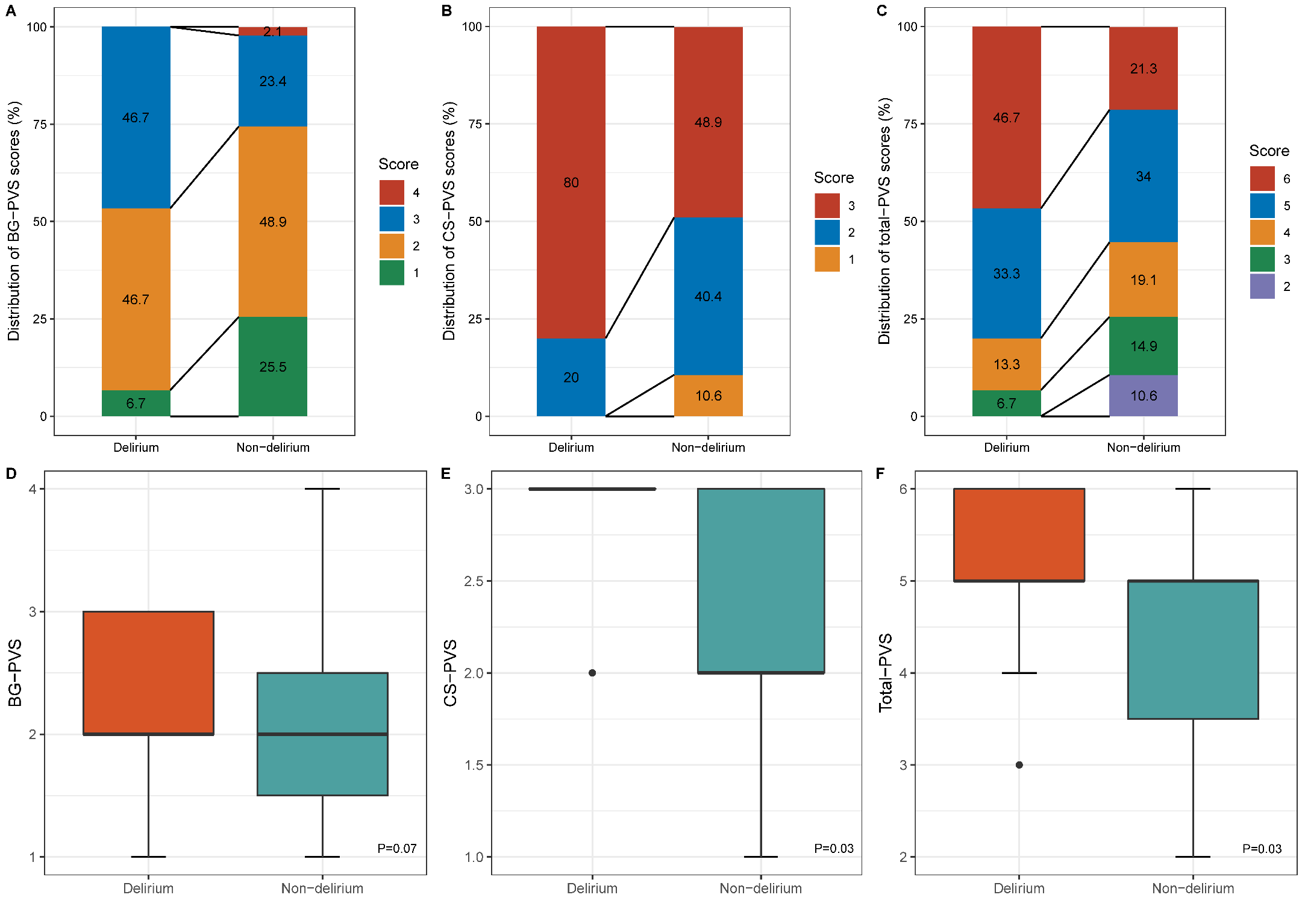

Supplement: Supplementary file 1 [file Data_Sheet_1.docx]
